# Supplementary material for: Cross-platform clinical proteomics using the Charité open standard for plasma proteomics (OSPP)
Source: Nat Commun. 2025 Dec 22;16:11377. doi: 10.1038/s41467-025-67264-9 (PMC12727829; doi:10.1038/s41467-025-67264-9)
Supplement: Supplementary file 1 — Supplementary Information [file 41467_2025_67264_MOESM1_ESM.pdf]

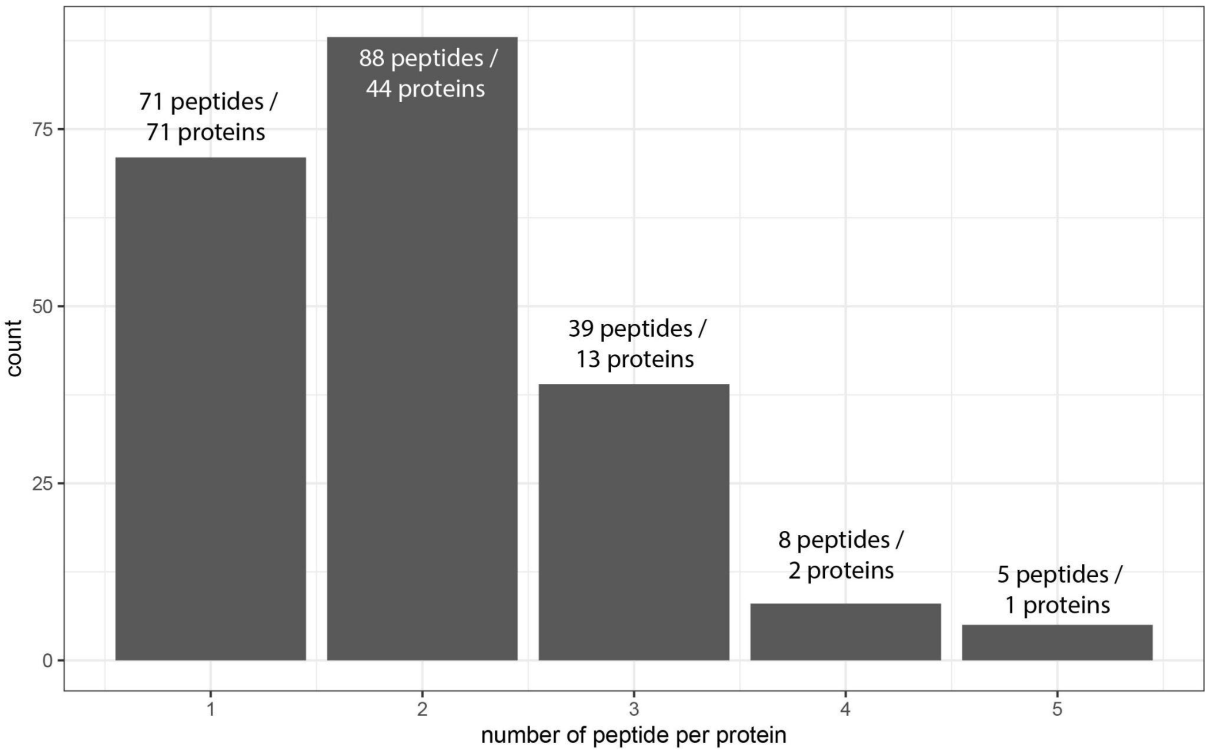

Supplementary Figure 1: Number of proteins grouped by the number of associated peptides. Bar plot showing the distribution of peptide coverage per protein. The x-axis represents the number of peptides assigned to each protein, and the y-axis indicates the number of proteins with that level of peptide coverage.

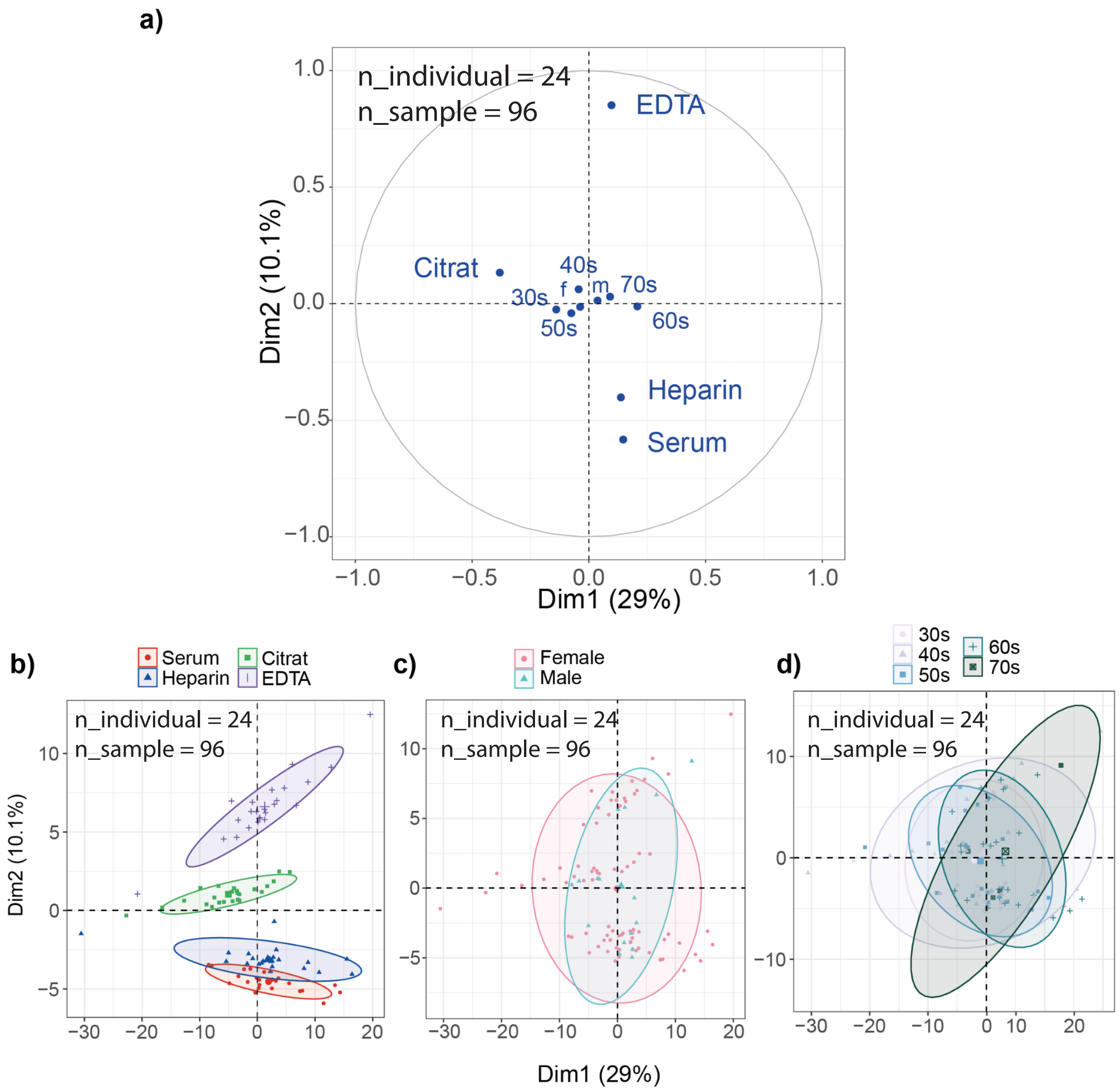

Supplementary Figure 2: Principal Component Analysis (PCA) of OSPP Peptide Quantification Across Blood Matrices and Patient Metadata

(a) Loading plot showing the contribution of individual peptides to the principal components (PC1 and PC2), based on normalized quantities of 197 consistently quantified OSPP peptides. PC1 (29%), while PC2(10.1%).

(b–d) Principal component analysis (PCA) plots of the same dataset, colored by:

(b) blood matrix type, (c) sex, and (d) age group.

Ellipses represent 80% confidence intervals around each group, highlighting the separation and clustering patterns driven by the respective variables. These plots demonstrate the analytical consistency of the OSPP peptide panel across both biological and technical conditions.

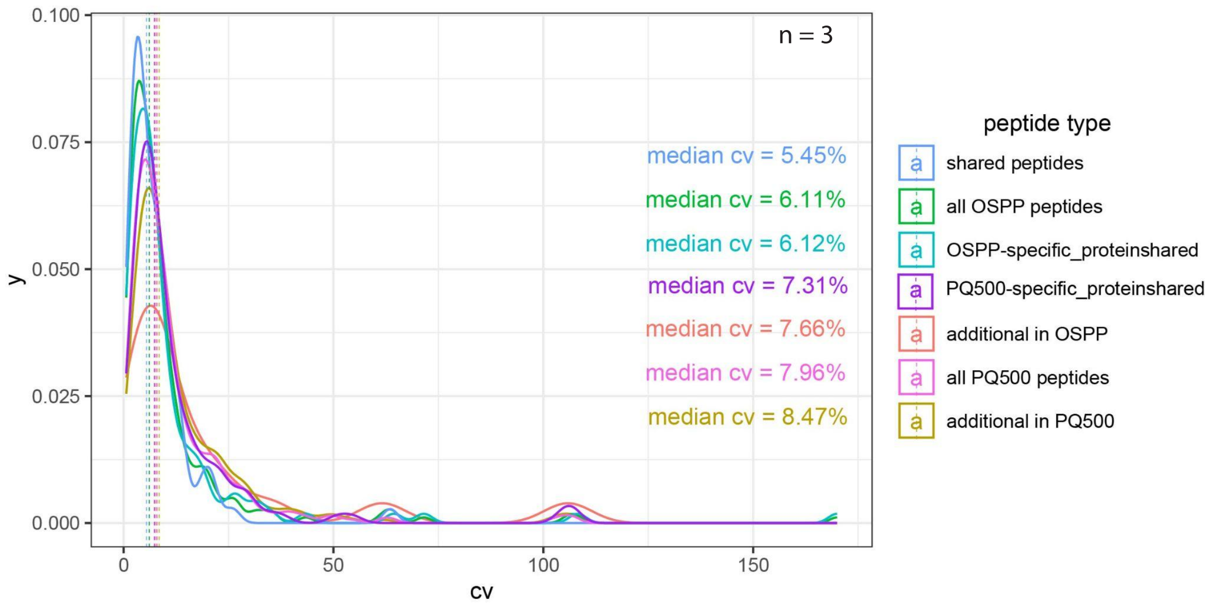

Supplementary Figure 3a
Comparison of Peptide CV Distributions Between OSPP and PQ500 Selections — Triplicate Injections of Plasma Pool on Micro-flow Attached ZenoTOF
Density plots show the distribution of peptide-level coefficients of variation (CV, calculate by Standard deviation / mean) for different peptide sets measured across triplicate measurements of plasma pool. Each curve represents a specific peptide group within a matrix. Lower CVs indicate higher analytical reproducibility in the study cohort. The peptide sets are defined as follows:

- **Shared peptides**: 81 peptides that are shared between the OSPP and PQ500 panels
- **All OSPP peptides**: 207 OSPP peptides consistently quantified quantified in all three replicates
- **OSPP-specific_proteinshared**: 108 OSPP peptides originating from proteins shared with PQ500, but having different peptide sequences
- **PQ500-specific_proteinshared**: 133 PQ500 peptides originating from proteins shared with OSPP, but having different peptide sequences
- **Additional in OSPP**: 18 peptides uniquely present in the OSPP panel
- **All PQ500 peptides**: 352 PQ500 peptides consistently quantified in all three replicates
- **Additional in PQ500**: 271 PQ500 peptides detected in the dataset but not included in the OSPP panel

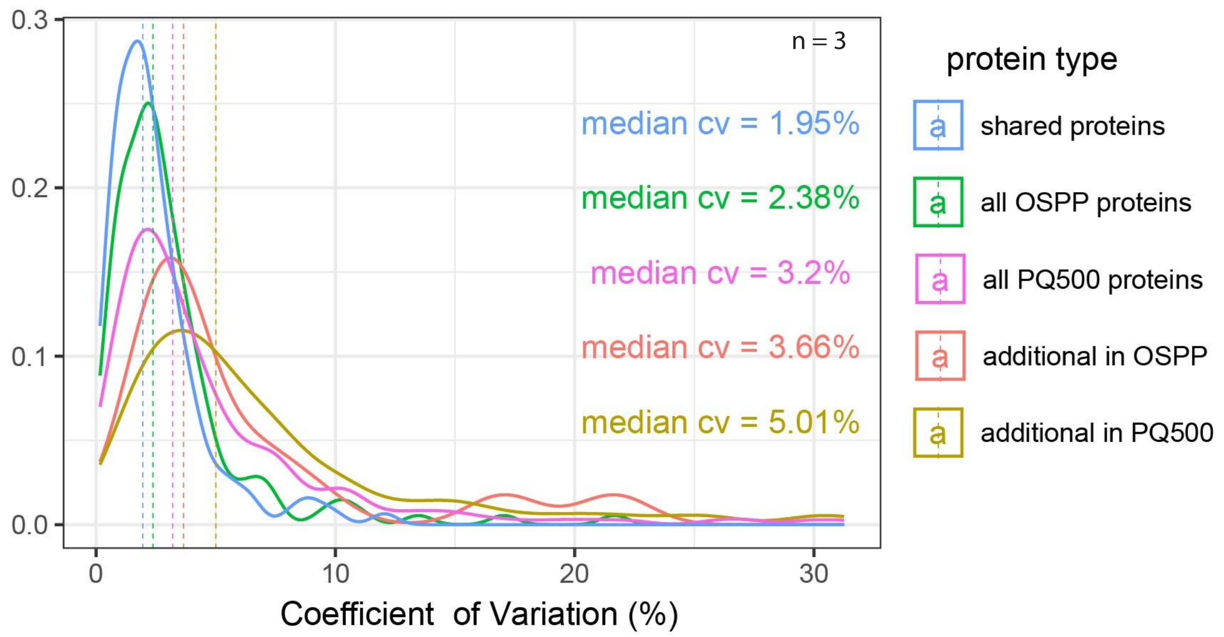

Supplementary Figure 3b

Comparison of Protein CV Distributions Between OSPP and PQ500 Selections — Triplicate Injections of Plasma Pool on Micro-flow Attached ZenoTOF

Density plots show the distribution of protein-level coefficients of variation (CV, calculate by Standard deviation / mean) for different peptide sets measured across triplicate measurements of plasma pool. Each curve represents a specific peptide group within a matrix. Lower CVs indicate higher analytical reproducibility in the study cohort. The protein sets are defined as follows:

- **Shared proteins**: 116 proteins that are shared between the OSPP and PQ500 panels
- **All OSPP proteins**: 128 OSPP proteins consistently quantified in more than two-thirds of the samples
- **All PQ500 proteins**: 213 PQ500 proteins consistently quantified in more than two-thirds of the samples
- **Additional OSPP proteins**: 15 immunoglobulins uniquely present in the OSPP panel
- **Additional PQ500 proteins**: 97 PQ500 proteins detected in the dataset but not included in the OSPP panel

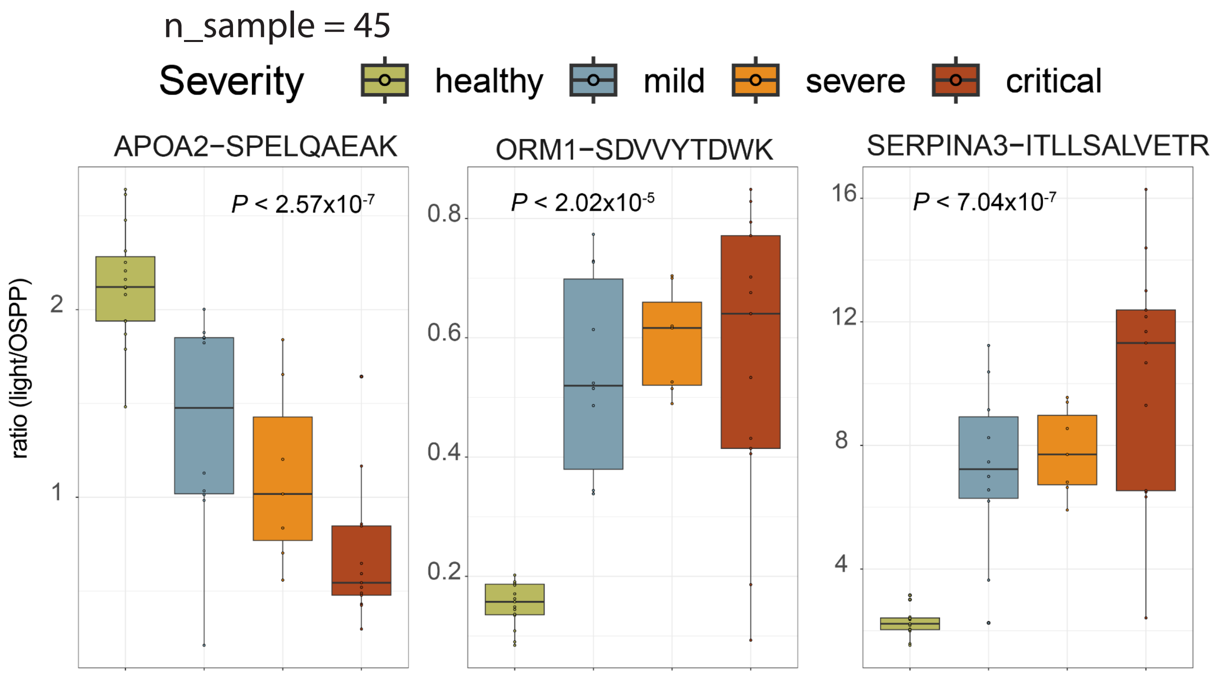

Supplementary Figure 4: **OSPP-Normalized Quantities of Representative Peptides Across Concentration Groups and Disease Severities _ MRM-HR**

Visualization of peptide-level responses to COVID-19 severity using MRM-HR. Selected peptides reflect proteins commonly shown to have severity-dependent trends and differentiate between healthy and COVID-19-infected individuals. Boxplots display OSPP-normalized peptide quantities across patient groups of increasing severity, as described in panel a. Boxes represent the 25th to 75th percentiles (with the median as a central line), and whiskers show the full data range excluding outliers. Extracted ion chromatograms illustrate representative peptide signals across severity categories. P-values were calculated using ordinal linear regression based on the COVID-19 treatment escalation score (healthy, mild, severe, critical) and adjusted for multiple testing using the Benjamini–Hochberg procedure

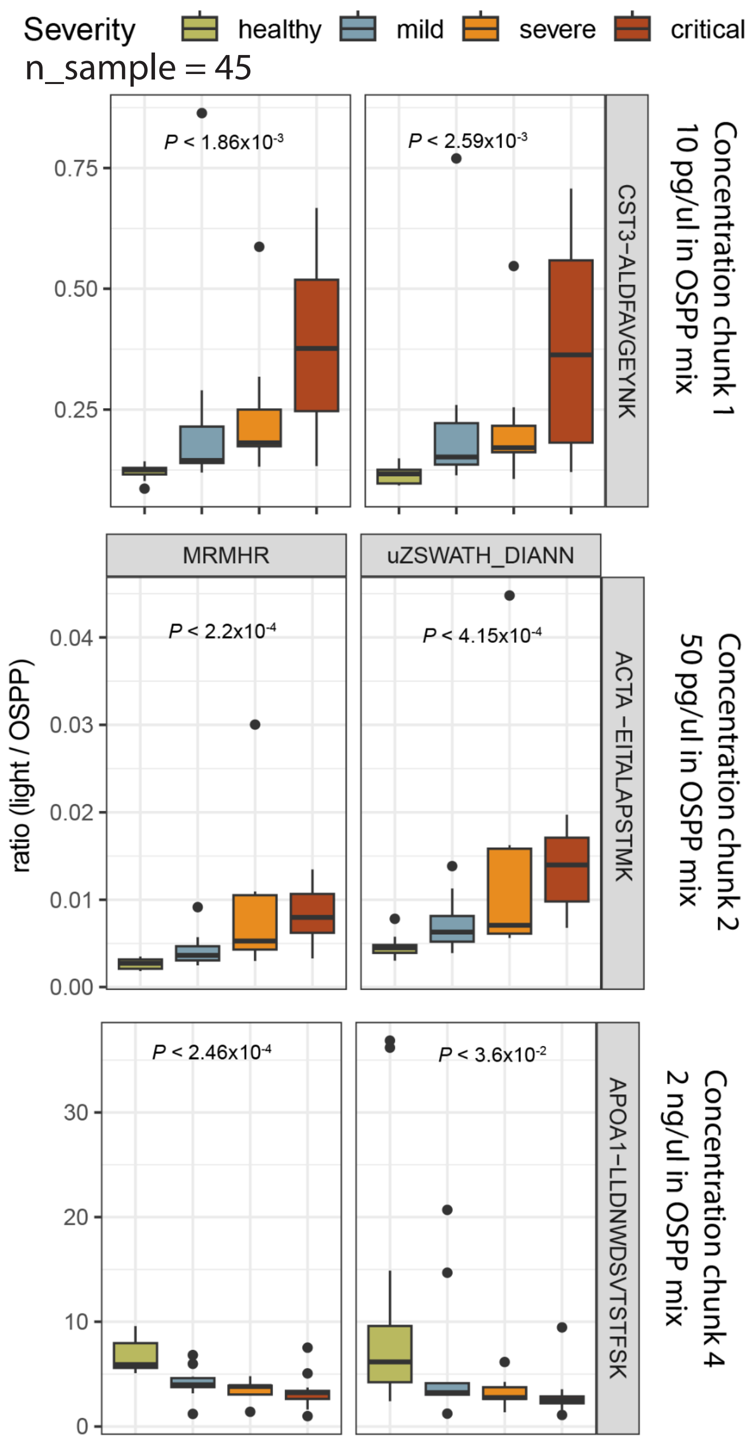

Supplementary Figure 5: **OSPP-Normalized Quantities of Representative Peptides Across Concentration Groups and Disease Severities_uzswath**

Visualization of peptide-level responses to COVID-19 severity using uzswath.
Boxplots show the normalized ("ratio") peptide quantities for representative peptides selected from different abundance groups within the OSPP panel, measured across patient samples stratified by clinical severity. Each peptide represents a distinct concentration range (10 pg/µl, 50 pg/µl, 2 ng/µl) within the overall panel. Boxes represent the 25th to 75th percentiles, with the median shown as a horizontal line; whiskers extend to the most extreme values within 1.5× the interquartile range. Outliers are excluded. The plots demonstrate the robustness of OSPP quantification across a wide

dynamic range and its ability to capture biologically meaningful variation across disease severity groups.

Supplementary Table 1 calculation formula for Zeno MRM-HR method

Charge State	Slope	Intercept
1	0.05	5
2	0.049	-1
3	0.048	-2
4	0.05	-2
