# Supplementary material for: Cross-platform clinical proteomics using the Charité open standard for plasma proteomics (OSPP)
Source: Nat Commun. 2025 Dec 22;16:11377. doi: 10.1038/s41467-025-67264-9 (PMC12727829; doi:10.1038/s41467-025-67264-9)
Supplement: Supplementary file 2 — Description of Additional Supplementary Files [file 41467_2025_67264_MOESM2_ESM.pdf]

Description of Additional Supplementary Files

File Name: Supplementary Data 1

Description:

peptide selection parameters, properties and analytical performance

Sheet name	Description
selection cohort	Cohort description for peptide selection
peptide properties	peptide selection parameters, properties, analytical performance and matrix test detection
Peptide quantities in different	pairwise Wilcoxon signed-rank tests for each peptide in every two blood matrices
pairwise Wilcoxon signed-rank t	pairwise Wilcoxon signed-rank tests for each peptide in every two blood matrices
CV_compare with PQ500_peptide	coefficient of variation in percentage (sd/mean) of each peptide (Precursor.Normalised) calculated from triplicate measurements of pooled plasma on micro-flow attached ZenoTOF
CV_compare with PQ500_protein	coefficient of variation in percentage (sd/mean) of each protein (MaxLFQ) calculated from triplicate measurements of pooled plasma on micro-flow attached ZenoTOF
PXD036594_ourput	DIA-NN ourput of PXD036594 (using OSPP pipline, filtered for OSPP/ PQ500 peptides)
PXD036594_CV_compare with PQ500	coefficient of variation in percentage (sd/mean) of each peptide (Precursor.Normalised) calculated from PXD036594

File Name: Supplementary Data 2

Description:

amount of each isotopic labeled peptide in 1µl of the concentration -matched OSPP mixture

Sheet name	Description
Sheet 1	amount of each isotopic labeled peptide in 1µl of the concentration -matched OSPP mixture

File Name: Supplementary Data 3

Description:

calibration curves & LOQs

Sheet name	Description
Preparation of Calibration curve	Preparation of Calibraiton curves, methods and concentration used
LOD,LOQ	Summary of limit of detection and quantification and fitted linear regression formula (slope, intercept and R2)

File Name: Supplementary Data 4

Description:

metadata for the manuscript

Sheet name	Description
metadata for 4 matrices	metadata for healthy individual cohort with 4 matrices
meta data for study cohort	Metadata for the COVID-19 plasma cohort.
WHO score & Severity	WHO score and severity description
metadata for exploris480 sample	Sample name reference for measurement on Exploris 480 (data match for nthermo-MS1 & -MS2)
Composite dataset	Composite dataset, in each severity group, randomly select data acquired from different LC-MS platforms and make the composite dataset

File Name: Supplementary Data 5

Description:

COVID-19 cohort_ MRMHR data

Sheet name	Description
fragment used for quantificaiton	Fragment used for quantificaiton in MRM-HR
Kendall´s Tau trend test_WHO	Statistical analysis of each peptide . two-sided Kendall´s Tau trend test between peptide quantitites and COVID19 treatment escalation score (WHO 0, 3, 4, 5, 6, 7)
Kendall´s Tau trend test_Severity	Statistical analysis of each peptide . Kendall´s Tau trend test between peptide quantitites and COVID-19 severity (healthy, mild,severe,critical)

File Name: Supplementary Data 6

Description:

fragment distribution, CV, sample fold change, R2, slope and Statistical analysis of each peptide acquired from MRMHR and ZenoSWATH-DIA data.

Sheet name	Description
Fragment distribution	distribution of each fragment (% compared to all fragment in the respective precursor) in each LC-MS platform
foldchange	fold change of peptide quantities of DIA (uZSWATH) generated from DIA-NN software to that of MRM-HR generated by skyline, calculated by uZSWATH quantities / MRMHR quantities (norm_light: median normalised value; ratio: ratio (light / OSPP))
CV for studypools (MADmedian)	coefficient of variation (MAD/median) in percentage of each peptide calculated from quintuplicates of study pools on both acquisition methods
CV for studypools (SDmean)	coefficient of variation (SD/mean) in percentage of each peptide calculated from quintuplicates of study pools on both acquisition methods
R2-slope	Correlation of each peptide calculated from linear model(y~x) of MRM-HR to ZenoSWATH-DIA (R2 and slope)
Kendall´s Tau trend_WHO	Statistical analysis of each peptide in both MS methods . two-sided Kendall´s Tau trend test between peptide quantitites and COVID19 treatment escalation score (WHO 0, 3, 4, 5, 6, 7)
Kendall´s Tau trend Severity	Statistical analysis of each peptide . Kendall´s Tau trend test between peptide quantitites and COVID-19 severity (healthy, mild,severe,critical)

File Name: Supplementary Data 7

Description:

OSPP peptide detection, RT, fragment distribution, CV, and Statistical analysis of each peptide acquired from all DIA platforms

Sheet name	Description
Detection of peptide in certain	Peptide detection in each WHO severity group under each acquisition platforms, numbers shown are the number of sample within each severity groups
Retention time of each sample	Retention time of each precursor and the difference between light and OSPP in all sample in each platforms
RT differnece between light & O	Retention time cv and range of both light and OSPP (heavy) peptides
Fragment distribution	distribution of fragment (% among all fragments in respective precursor) of each peptide in each platform
icc fragment percentage of all	The resulting output data file is a structured table summarizing measurement variability and reproducibility metrics for every precursor–fragment–label combination in the dataset.
correlation between peptides fr	R2 of peptides from same protein in different DIA platform calculated from linear model(y~x)
Precurs used for quantificaiton	Precursor used for quantification in each LC-MS platforms
CV for studypools(MADmedian)	coefficient of variation (MAD/median) in percentage of each peptide calculated from quintuplicates of study pools on all platforms
CV for studypools(SDmean)	coefficient of variation (SD/mean) in percentage of each peptide calculated from quintuplicates of study pools on all platforms
CV for all samples(MAdmedian)	coefficient of variation (MAD/median) in percentage of each peptide calculated from clinical samples on all platforms
CV for all samples(SDmean)	coefficient of variation (SD/mean) in percentage of each peptide calculated from clinical samples on all platforms
variance partitioning across pe	Variance Partitioning of Peptide-Level Expression (ratio: OSPP normalised and norm_light: median normalised peptide quantities) Across MS Platform and Disease Severity

Kendall´s Tau trend test_WHO	Statistical analysis of each peptide ineach platform . two-sided Kendall´s Tau trend test between peptide quantitites and COVID19 treatment escalation score (WHO 0, 3, 4, 5, 6, 7)
Kendall´s Tau trend test_Severi	Statistical analysis of each peptide . two-sided Kendall´s Tau trend test between peptide quantitites and COVID-19 severity (healthy, mild,severe,critical)
composite dataset composition	Samples contribute to composite dataset
Statistics_composite dataset	Statistical analysis of each peptide in both ZT Scan DIA dataset and composite dataset . two-sided Kendall´s Tau trend test between peptide quantitites and COVID-19 severity (healthy, mild,severe,critical)
bland-altman output	bland-altman output for comparing hZsSWATH to "composite dataset"
