# Supplementary material for: Cross-platform clinical proteomics using the Charité open standard for plasma proteomics (OSPP)
Source: Nat Commun. 2025 Dec 22;16:11377. doi: 10.1038/s41467-025-67264-9 (PMC12727829; doi:10.1038/s41467-025-67264-9)
Supplement: Supplementary file 4 — Reporting Summary [file 41467_2025_67264_MOESM4_ESM.pdf]

nature portfolio

Corresponding author(s): Markus Ralser

Last updated by author(s): Nov 17, 2025

Reporting Summary

Nature Portfolio wishes to improve the reproducibility of the work that we publish. This form provides structure for consistency and transparency in reporting. For further information on Nature Portfolio policies, see our Editorial Policies and the Editorial Policy Checklist.

Statistics

For all statistical analyses, confirm that the following items are present in the figure legend, table legend, main text, or Methods section.

n/a	Confirmed	
☐	☒	The exact sample size (*n*) for each experimental group/condition, given as a discrete number and unit of measurement
☐	☒	A statement on whether measurements were taken from distinct samples or whether the same sample was measured repeatedly
☐	☒	The statistical test(s) used AND whether they are one- or two-sided<br>*Only common tests should be described solely by name; describe more complex techniques in the Methods section.*
☐	☒	A description of all covariates tested
☐	☒	A description of any assumptions or corrections, such as tests of normality and adjustment for multiple comparisons
☐	☒	A full description of the statistical parameters including central tendency (e.g. means) or other basic estimates (e.g. regression coefficient) AND variation (e.g. standard deviation) or associated estimates of uncertainty (e.g. confidence intervals)
☐	☒	For null hypothesis testing, the test statistic (e.g. *F*, *t*, *r*) with confidence intervals, effect sizes, degrees of freedom and *P* value noted<br>*Give P values as exact values whenever suitable.*
☒	☐	For Bayesian analysis, information on the choice of priors and Markov chain Monte Carlo settings
☒	☐	For hierarchical and complex designs, identification of the appropriate level for tests and full reporting of outcomes
☐	☒	Estimates of effect sizes (e.g. Cohen's *d*, Pearson's *r*), indicating how they were calculated

Our web collection on statistics for biologists contains articles on many of the points above.

Software and code

Policy information about availability of computer code

Data collection	The raw proteomics data from all DIA methods was processed using DIA-NN, 1.8.1 obtained from GitHub, detailed pipeline and raw data were provided in Mendeley<br>Zeno MRM-HR data were processed using Skyline (64-bit, v.23.1.0.268), output are provided in supplementary table
Data analysis	Wilcoxon signed-rank test<br>To assess whether the relative abundance rankings of peptides differ across blood matrices, we performed pairwise Wilcoxon signed-rank tests for each peptide. For each comparison, peptide intensity values from matched samples (same donor measured across matrices) were tested using the wilcox.test() function in R (v4.2.2) with paired = TRUE. All six pairwise matrix combinations were tested independently. To account for multiple hypothesis testing, p-values were adjusted using the Benjamini-Hochberg (BH) procedure with the p.adjust(method = "BH") function. The resulting adjusted p-values were compiled into a long-format data table with peptide identifiers and matrix comparison labels. Peptides showing significant rank shifts (adjusted p < 0.05) were flagged for further interpretation.<br>coefficient of variation (CV)<br>To assess intra-group variability of peptide precursors, we calculated the coefficient of variation (CV) using two methods. The primary method employed a robust measure, defined as the median absolute deviation (MAD) divided by the median of intensity values, expressed as a percentage (CV = MAD / Median × 100). This approach was implemented using the mad() function from the R stats package (v4.2.2), offering robustness to outliers and suitability for skewed or heteroscedastic data commonly observed in proteomics. For comparison, we also computed CV using the conventional formula based on standard deviation divided by the mean (CV = SD / Mean × 100). Both measures are reported where relevant to provide complementary insights into variability.<br>Kendall's tau (KT) trend test

Significance testing of the trend between peptide quantities (normalized endogenous quantity or ratio) and the ordinal classification as provided by the WHO disease severity (levels as indicated) was performed using Kendall's tau (KT) statistics as implemented in the "EnvStats v2.8.1" R package "kendallTrendTest" function. For the clinical cohort, the KT statistics were calculated as the trend of absolute peptide concentrations against the following WHO groups: 0, 3, 4, 5, 6, and 7; selected peptides in each comparison were used for data analysis, without imputation. Where indicated, multiple testing correction was performed by controlling for false discovery rate using the Benjamini-Hochberg procedure 1 as provided by the R package "stats v4.2.2" - "p.adjust" function. A full summary of these statistical test results is provided in the respective supplementary tables. (Adjusted) p-values were considered significant when $p < 0.05$.

Ordinal logistic regression

Significance testing of the association between absolute peptide concentrations and clinical disease severity was performed using ordinal logistic regression, as implemented in the MASS v7.3-61 R package via the polr (proportional odds logistic regression) function. For clinical cohort analysis, disease severity was modeled as an ordered categorical variable, based on WHO clinical classification grouped into four categories: healthy, mild, moderate, and critical. These categories were derived from WHO levels as follows: healthy (WHO 0), mild (WHO 3), moderate (WHO 4–5), and critical (WHO 6–7). The model estimated the association between absolute peptide concentrations and increasing disease severity under the proportional odds assumption. No imputation was applied; only peptides with observed intensity values were included. Where applicable, p-values were adjusted for multiple testing using the Benjamini-Hochberg procedure to control the false discovery rate, as implemented in the stats v4.2.2 R package via the p.adjust function. A full summary of model coefficients and statistical test results is provided in the respective Supplementary Tables. Adjusted p-values were considered significant when $p < 0.05$.

Variance Partitioning Analysis

To assess the contribution of technical and biological factors to peptide-level variability, we used the variancePartition R package. A linear mixed-effects model was applied with MS platform (MS) and disease severity (Severity) as random effects, using the formula:

expression ~ (1 | MS) + (1 | Severity)

Missing values are imputed with zero. The model was fitted using fitExtractVarPartModel() from the variancePartition v1.36.2 R package, and the proportion of variance explained by each factor was computed for each peptide. The results were summarized to quantify the overall impact of technical and biological variance in the dataset.

Bland–Altman Analysis

To assess the agreement between different mass spectrometry acquisition methods, we performed Bland–Altman analyses at the peptide level (using ratios in two datasets). For each experimental condition, peptides were filtered to exclude infinite values and missing data. For every peptide detected under each condition, the mean and difference of the ratios from two datasets (e.g., hZsSWATH dataset and the composite dataset (not including any data from hZsSWATH dataset to avoid data leakage)) were calculated. The bias (mean difference), standard deviation (SD), and limits of agreement (LoA, calculated as bias ± 1.96 × SD) were determined. Confidence intervals (95%) for the bias and LoA were estimated using Student's t-distribution and the standard errors derived from the sample variance and size. Bland–Altman plots were generated for each peptide, visualizing the agreement between methods along with the calculated LoA and confidence intervals. The corresponding summary statistics were compiled into Supplementary table 7. All calculations and visualizations were performed in R using custom scripts based on the tidyverse (v 2.0.0) package for data handling and base R graphics functions for plotting.

For manuscripts utilizing custom algorithms or software that are central to the research but not yet described in published literature, software must be made available to editors and reviewers. We strongly encourage code deposition in a community repository (e.g. GitHub). See the Nature Portfolio guidelines for submitting code & software for further information.

Data

Policy information about availability of data

All manuscripts must include a data availability statement. This statement should provide the following information, where applicable:

- Accession codes, unique identifiers, or web links for publicly available datasets
- A description of any restrictions on data availability
- For clinical datasets or third party data, please ensure that the statement adheres to our policy

The raw proteomics data generated in this study have been deposited in ProteomeXchange with identifier PXD070765 (https://www.ebi.ac.uk/pride/archive/projects/PXD070765). The processed proteomics data are available at Mendeley - Proteomics Data (DIA-NN & skyline). The data generated in this study are provided in the Supplementary Data.

Research involving human participants, their data, or biological material

Policy information about studies with human participants or human data. See also policy information about sex, gender (identity/presentation), and sexual orientation and race, ethnicity and racism.

Reporting on sex and gender	No sex or gender specific analyses were performed as part of this technical validation study.
Reporting on race, ethnicity, or other socially relevant groupings	The cohort has been described before (Wang et al 2022, https://doi.org/10.1016/j.eclinm.2022.101495). This analysis includes no data on race, ethnicity, or other socially relevant groupings.
Population characteristics	The cohort has been described before The cohort has been described before (Wang et al 2022, https://doi.org/10.1016/j.eclinm.2022.101495).n brief, the cohort comprised 10/30 (33%) women and the overall median age was 62 years (IQR: 50-37).
Recruitment	Patients were recruited within the Pa-COVID-19 study conducted at Charité - Universitätsmedizin Berlin, a prospective observational cohort study on the pathophysiology of COVID-19. All patients with PCR-confirmed SARS-CoV-2 infection were eligible for inclusion. Refusal to provide informed consent by the patient or a legal representative and any condition prohibiting supplemental blood collection for serial biosampling were exclusion criteria. Patients were treated according to current national and international guidelines. The cohort has been described before (Wang et al 2022, https://doi.org/10.1016/j.eclinm.2022.101495).

Ethics oversight

The COVID-19 cohort is a subcohort of the Pa-COVID-19 study conducted at Charité - Universitätsmedizin Berlin, Germany, and the matrix test cohort is part of prospective observational cohort study. Both studies were carried out in accordance with the Declaration of Helsinki and the principles of Good Clinical Practice (ICH 1996), where applicable, and were approved by the ethics committee of Charité – Universitätsmedizin Berlin (EA2/066/20, EA4/245/20). Written informed consent was obtained from all participants or their legal guardians before initiation of study procedures. All data have been de-identified and presented in aggregate, with age reported as ranges to prevent identification, while maintaining sex/gender reporting in accordance with journal policy.

Note that full information on the approval of the study protocol must also be provided in the manuscript.

Field-specific reporting

Please select the one below that is the best fit for your research. If you are not sure, read the appropriate sections before making your selection.

[x] Life sciences   [ ] Behavioural & social sciences   [ ] Ecological, evolutionary & environmental sciences

For a reference copy of the document with all sections, see nature.com/documents/nr-reporting-summary-flat.pdf

Life sciences study design

All studies must disclose on these points even when the disclosure is negative.

Sample size

45 human samples were used for the study

Data exclusions

The completeness of data for each peptide was evaluated based on its frequency of detection across all biological samples. Peptides were considered if they were detected in more than 66.7% (⅔) of the samples. We calculated the percentage of each peptide measured on each LC-MS platform/method and exclude the peptides with a completeness value less than 66.7% for subsequent analysis.

Replication

plasma samples were measured one time on each platform, quality control samples (pool of all studies) were measured 3 times on each platform. for calibration curves to check LOQs, each calibration curves were measured 3 times on each platform

Randomization

the samples were analysed in a fully randomised way on each LC-MS platform

Blinding

blinding is applied during data acquisition, process and pre-processing of data (data exclusion and batch correction)

Reporting for specific materials, systems and methods

We require information from authors about some types of materials, experimental systems and methods used in many studies. Here, indicate whether each material, system or method listed is relevant to your study. If you are not sure if a list item applies to your research, read the appropriate section before selecting a response.

Materials & experimental systems

n/a	Involved in the study	
[x]	[ ]	Antibodies
[x]	[ ]	Eukaryotic cell lines
[x]	[ ]	Palaeontology and archaeology
[x]	[ ]	Animals and other organisms
[x]	[ ]	Clinical data
[x]	[ ]	Dual use research of concern
[x]	[ ]	Plants

Methods

n/a	Involved in the study	
[x]	[ ]	ChIP-seq
[x]	[ ]	Flow cytometry
[x]	[ ]	MRI-based neuroimaging

Plants

Seed stocks

Report on the source of all seed stocks or other plant material used. If applicable, state the seed stock centre and catalogue number. If plant specimens were collected from the field, describe the collection location, date and sampling procedures.

Novel plant genotypes

Describe the methods by which all novel plant genotypes were produced. This includes those generated by transgenic approaches, gene editing, chemical/radiation-based mutagenesis and hybridization. For transgenic lines, describe the transformation method, the number of independent lines analyzed and the generation upon which experiments were performed. For gene-edited lines, describe the editor used, the endogenous sequence targeted for editing, the targeting guide RNA sequence (if applicable) and how the editor was applied.

Authentication

Describe any authentication procedures for each seed stock used or novel genotype generated. Describe any experiments used to assess the effect of a mutation and, where applicable, how potential secondary effects (e.g. second site T-DNA insertions, mosiacism, off-target gene editing) were examined.
